# florio HAEMO—A Digital Medical Device for Monitoring of Treatment, Symptoms and Physical Activities for People Living With Haemophilia

**DOI:** 10.1111/hae.70198

**Published:** 2026-01-13

**Authors:** Christoph Königs, Jan Astermark, Jan Blatny, Jamie O´Hara, Allfonso Iorio, Claude Negrier, Flora Peyvandi, Katharina Steinitz, Armin J. Reininger, Santiago Bonanad

**Affiliations:** ^1^ Goethe University, University Hospital, Department of Paediatrics and Adolescent Medicine, Clinical and Molecular Haemostasis Frankfurt Germany; ^2^ Institution of Translational Medicine and Department of Hematology, Oncology and Radiation Physics Lund University, Skåne University Hospital Malmö Sweden; ^3^ Department of Pediatric Hematology and Biochemistry University Hospital Brno and Faculty of Medicine of Masaryk University Brno Czech Republic; ^4^ HCD Economics Daresbury UK; ^5^ University of Chester Chester UK; ^6^ Department of Health Research Methods, Research and Impact McMaster University Hamilton Ontario Canada; ^7^ Department of Medicine McMaster University Hamilton Ontario Canada; ^8^ UR 4609 Hemostasis & Thrombosis Lyon 1 University Lyon France; ^9^ Angelo Bianchi Bonomi Hemophilia and Thrombosis Center Fondazione IRCCS Ca' Granda Ospedale Maggiore Policlinico Milan Italy; ^10^ Department of Pathophysiology and Transplantation Università Degli Studi di Milano Milan Italy; ^11^ Florio GmbH Munich Germany; ^12^ Ludwig‐ Maximilians‐University Munich Munich Germany; ^13^ Hospital Universitari i Politècnic La Fe Valencia Spain

**Keywords:** digital medical device, e‐diary, electronic patient diary, florio HAEMO, haemophilia, web‐based application

## Abstract

**Introduction:**

Despite therapeutic achievements in haemophilia care, there is still the need to monitor and define personal treatment outcomes and document results to achieve the best possible care. Hence, a need for unbiased, timely and comprehensive real‐world information exists to support informed shared decision‐making regarding treatment and care.

**Aim:**

To describe a medical device for people living with haemophilia (PLWH) supporting an active involvement to achieve a near to normal life.

**Methods:**

Florio HAEMO was developed as haemophilia monitoring platform to support PLWH and their care teams in documenting, interpreting and analysing personal reported outcomes. The tool was created partnering closely with PLWH and healthcare professionals to address previously unmet needs compared to existing applications.

**Results:**

Florio HAEMO was launched in March 2020. Currently, it is available in 25 countries and 24 languages; 1558 PLWH (86% with haemophilia A) are registered users in 121 treatment centres across 20 countries. All users included are on a prophylactic treatment regimen.

**Conclusion:**

Florio HAEMO allows the collection of contemporaneous data to monitor treatment, like factor level, adherence and consumption as well as monitoring treatment outcomes, including pain, bleeds, wellbeing and levels of physical activity to support self‐management, shared decision‐making and to enable better care for PLWH. Data collected over time may help to show the impact of individualised prophylaxis and may support the definition of factor levels required for good bleed and joint protection in a real world setting from daily life to physical activities.

## 1 Introduction

Haemophilia is a rare X‐linked congenital bleeding disorder characterised by a deficiency of coagulation factor VIII (FVIII) in haemophilia A (HA) or factor IX (FIX) in haemophilia B (HB) as a result of pathogenic variants in the *F8* and *F9* clotting factor genes [1]. Core to the natural course of haemophilia are bleeds including joint haemorrhage, which may lead to arthropathy and disability. Recommended as an effective way for restoring haemostasis to prevent bleeds is prophylaxis with clotting factor concentrates (CFC) or non‐factor therapies [1]. Modern treatment has changed perspectives and possibilities for participation in social and physical activities as well as enabling different lifestyles, allowing for jobs that were not recommended in the past. Given the general benefits of physical activity and sports on overall health, and also in light of increasing prevalence of overweight and obesity in people living with haemophilia (PLWH), individualised haemophilia treatment targeting bleed prevention accordingly to the desired level of physical activity has been the preferred choice [2]. Individualised treatments render differences between individuals, making documentation of treatments and outcomes becomes a real need.

### 1.1 Current Treatment Goals and Existing Unmet Need

Treatment goals for haemophilia changed significantly over the last decades, from survival and reduction of sequelae to achieving zero bleeds, health equity and normalisation of haemostasis [3]. While current available medications and prophylaxis approaches clearly show dramatic improvements with respect to annualised bleeding rates (ABR) over the last decades, recent data show that even early prophylaxis does not fully prevent arthropathy with subclinical bleedings and changes in joint magnetic resonance imaging being reported as early as in adolescence even in patients undergoing high intensity prophylaxis [4]. In addition, the MIND study from three Nordic countries revealed that there is still consistent higher use of medication for pain, depression, and anxiety and even an increased presence of comorbidities not related to haemophilia and mortality among PLWH compared with population controls [5]. These findings in adults are seen irrespectively of age or factor consumption.

Consequently, despite major improvement of modern treatment modalities, the next frontier for treatment is to enable PLWH to lead healthy and active lives [3], while preserving joint health. Better understanding of the pathophysiological mechanisms of joint diseases suggests that not only avoidance of clinically overt bleeds but moreover of subclinical bleeds into joints, and thereby triggered synovitis, may be the next important level of treatment goals. Physical activity and exercise have been demonstrated to improve muscle strength, flexibility, balance as well as quality of life and may even improve bleeding tendency [6, 7, 8, 9, 10].

Several attempts have been made to identify minimal factor level requirements to prevent bleeding episodes, maintain joint health, avoid synovitis and allow an active lifestyle. The *World Federation of Hemophilia* (WFH) strongly recommends trough levels of at least 3%–5% based on bleeding rates in people with moderate or mild haemophilia only and an individualised prophylaxis in consideration of the bleeding phenotype, joint status, individual pharmacokinetics as well as self‐assessment and lifestyle preference of the individual [1]. There are no data available on required factor levels to perform different physical activities safely, so far recommendations are mainly based on expert opinion [11, 12] and personal experience and safe levels may vary significantly between individuals [13].

Thus, there is a critical need for unbiased, contemporaneous, and comprehensive real‐world information to allow well informed shared‐decision‐making regarding treatment and care on an individual level; especially in an era of new treatment approaches and new treatment goals striving for activity, health equity and a normal participation in life.

### 1.2 Electronic Diaries and Haemophilia

Treatment documentation as well accurate information on the efficacy of bleeding prevention, bleed treatment, type of bleeds and pain among other factors is required to provide the best possible care. Furthermore, accurate documentation represents a local requirement to obtain treatment in many countries. Electronic diaries have been developed mainly to aid documentation of treatments and reporting of bleeding episodes; examples include Smart Medication [14], Haemoassist [15, 16, 17, 18] and Haemtrack [16, 19]. Improvements in documentation quality and treatment compliance have been demonstrated through use of such electronic treatment diaries [17, 18]. Other tools have been developed to aid personalised PK‐guided prophylaxis (WAPPS‐HEMO [20], myWAPPS [21], myPKFiT [22, 23, 24]).

National and international haemophilia registries exist across Europe and worldwide [25, 26, 27], however, no standardised principles on the granularity of collected data have been implemented. In addition, interconnectivity between them and possibilities for combined data analysis are limited. Furthermore, systematic and standardised data collection in databases tailored to haemophilia and bleeding disorders is important for patient care on an individual but also on a group level. Data collected via electronic diaries could be shared with available national and international registries.

Nevertheless, there is still an unmet need for interoperability in the haemophilia data sources (registries, apps data, etc.) to obtain a uniform dataset to establish an interconnection of information via direct links or confederated approaches with the aim for a common dataset across databases [28]. Experts have already formulated that countries’ inability to report consistent and coherent data remains a challenge and hinders both provision of treatment and care for PLWH as well as optimal national and European healthcare systems [28]. With so many different systems available, patient metrics should be made easily available to the community of PLWH and their caregivers, especially in compliance with data protection and confidentiality in the current world of big data analysis [29].

Improvement of haemophilia care requires more than medication and simple documentation of events, but rather the use of multiple source data including patient reported data, such as treatments, adherence and treatment outcomes (bleeds, pain, etc.) presented as real time association of relevant information, such as factor levels during physical activity and respective outcomes, in order to achieve health equity and a participation in regular live. This report describes a medical device to support PLWH to achieve health equity and an active involvement to live a near to normal life with haemophilia.

## 2 Methods

### 2.1 Development of a New Monitoring Platform for People Living With Haemophilia

Florio HAEMO (Florio GmbH, Munich/Germany; https://florio‐haemo.com/) is a haemophilia monitoring platform for adults, adolescents, children and caregivers, developed to support PLWH and their care teams in documenting, interpreting and analysing personal reported outcomes. It was especially created to address the previously unmet needs by providing several novel features compared with some existing apps, aiming to support shared decision‐making and ultimately to enable better care for PLWH [30].

The platform was developed in close partnership with PLWH and healthcare professionals (HCP) from several European countries. During the development process, feedback from PLWH and HCP was initially requested by interviews, advisory boards and—depending on the state of platform development—by user testing and market research.

Several rounds of interviews as well as market research were conducted to understand the unmet needs of the haemophilia community. Real time data capture, sufficient level of detail, smart design to enable interpretation of information in the limited time to prepare before consultations as well as consistent data analysis across centres and countries and interoperability between databases were the key desirables by HCP identified during the process. PLWH preferred a digital solution, able to store all information in one place, showing current pharmacokinetic data adding value to the user. In addition, PLWH and HCPs acknowledged the chance to provide best possible individual care as well as improvement haemophilia care overall.

As the product evolves, updates are continuously made based on co‐development of PLWH and HCP to meet the needs and requirements of both user groups.

### 2.2 System Design

The system is designed as a cloud‐based platform, consisting of a smartphone app for PLWH and a web‐based dashboard for HCP. florio HAEMO app was designed using state‐of‐the‐art technology to support iOS and Android operating systems to ensure PLWH can run the app on their existing smartphones. The dashboard can be used on a standard web‐browser to enable HCP to see captured data in real time. In order to use florio HAEMO, HCPs need to register with florio HAEMO to activate a specific account for their haemophilia treatment centre. Access can be given to multiple HCPs in the same treatment centre. HCPs can invite PLWH in person or directly from the HCP dashboard. PLWH register directly via the florio HAEMO app. florio HAEMO was also designed to be able to interact with commercially available wearable devices and fitness trackers via their native phone health application.

Florio HAEMO is a CE‐marked medical device Class IIa under the EU Medical Regulation 2017/745.

### 2.3 Platform Features

Florio HAEMO is intended to monitor HA and HB therapy, for which it consists of the florio HAEMO smart phone app and the florio HAEMO web‐based dashboard. It offers to collect contemporaneous data on factor levels, pain levels, bleed details, joint assessments, well‐being, physical activity, as well as injection frequency and information on different treatment regimens and medication including prophylaxis with factor and non‐factor products, on demand treatment and ITI. florio HAEMO calculates data on treatment adherence, and factor consumption. Adherence is calculated based on the number of expected infusions according to the specified treatment plan in relation to the actually entered prophylactic injections within a time window of ‐ 4 h and +12 h based on the expected injection time. florio HAEMO also allows to integrate and correlate physical activity data, such as steps, active minutes, and so forth, derived from different sources like smartphones, health trackers, smartwatches and other wearable devices. Moreover, florio HAEMO is open to all available factor and non‐factor therapies licensed in the individual countries for haemophilia A or B.

**FIGURE 1:**
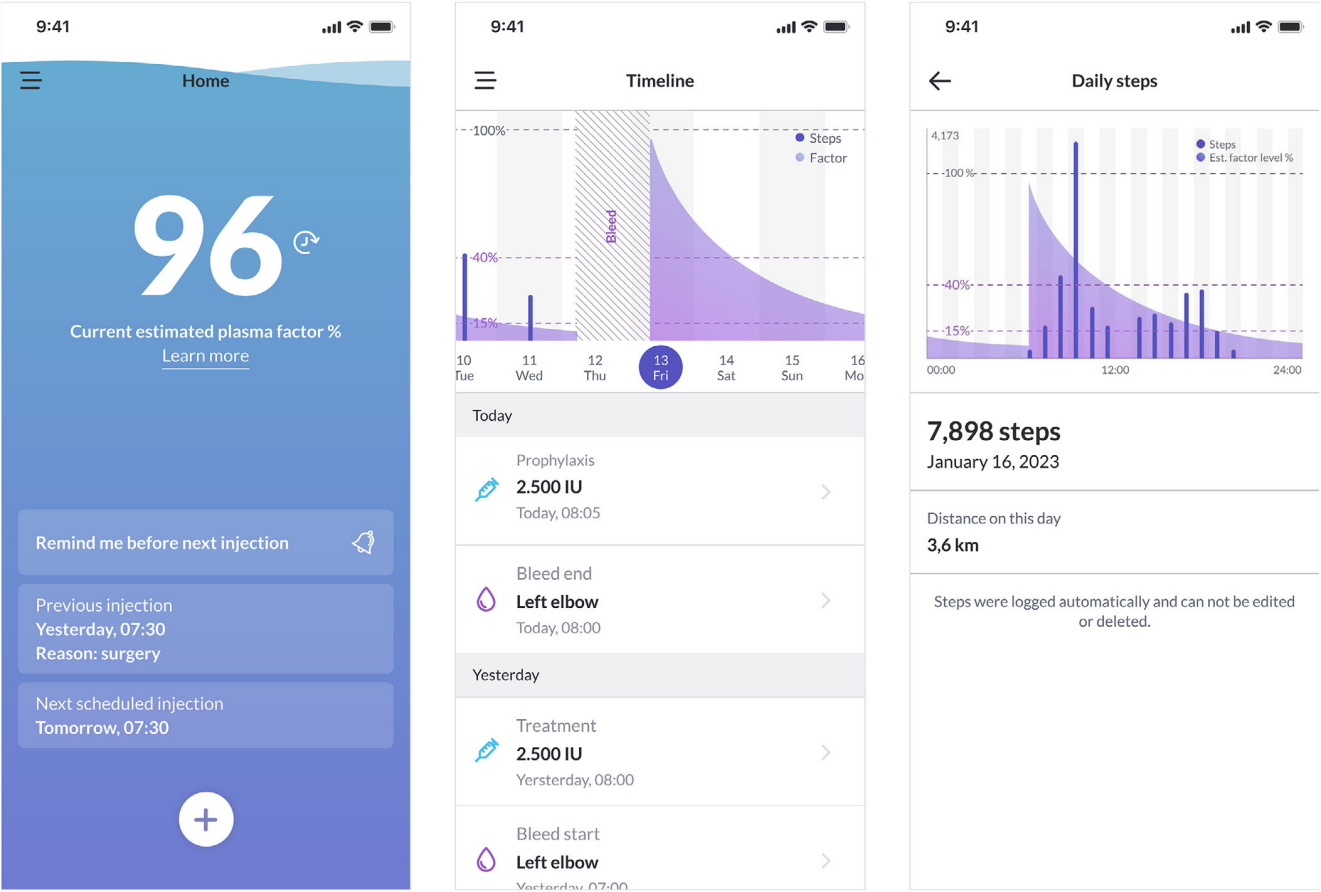
Screenshots of the florio HAEMO App (examples)—Home screen showing the current estimated factor level as well as previous and next injection. Timeline screen with details on entered data as well as correlation of estimated factor level with activity data. Activity data detail screen showing hourly steps in correlation to estimated factor level as well as total steps and corresponding distance. All information shown is exemplary data only, showing version 4.1. https://florio.com/florio‐haemo/ (accessed March 26th, 2025).

In addition, florio HAEMO accounts can be connected to McMaster PopPK [31], a CE‐marked device allowing estimation of individual PK profiles for a specific CFC, optimisation of an individual treatment regimen and forecasting of individual's factor level by Bayesian prediction during prophylaxis following factor administration [20]. After providing information on the individual PK profile and planned treatment schedule (dose and frequency), the app displays the current estimated factor level as well as predicted values until the next planned injection allowing PLWH to plan future daily activities based on entered prophylaxis details. Figure 1 shows an overview of the app features. The dashboard summarises all data entered, the factor level estimations as well as the captured activity data in a graphic format enabling the HCP to see documented treatment over time as well as details to each reported event. Figure 2 shows an example of the dashboard data visualizations.

FIGURE 2(a) Examples of florio HAEMO Dashboard Screens—Treatment Timeline: The treatment timeline shows summarized contextualised information of reported events, like injections, bleeds, pain, steps and physical activity over time. The monthly view shows information in more detail, for example, the estimated factor level over time. All information shown is exemplary data only, showing version 4.1. (b) *Example of florio HAEMO Dashboard Screen—Bleeds and pain*: Reported bleeds and pain events, their location on the body and a summary on the reported bleed causes are shown. The size of the circle on the body represents the amount of bleeds /pain reported in a specific location. All data entered via the app are shown with the associated event. Joint bleeds or target joint bleeds are highlighted automatically and ABR is calculated on the dashboard. All information shown is exemplary data only, showing version 4.1.

**Figure:**
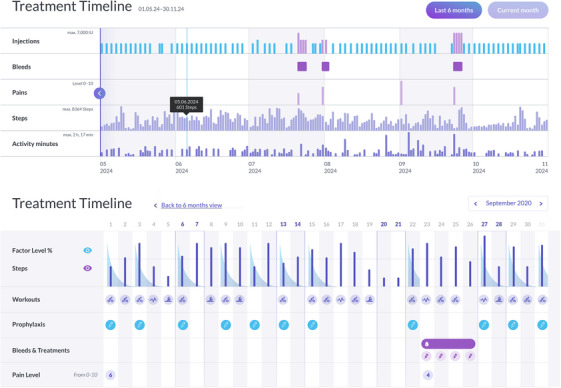

**Figure:**
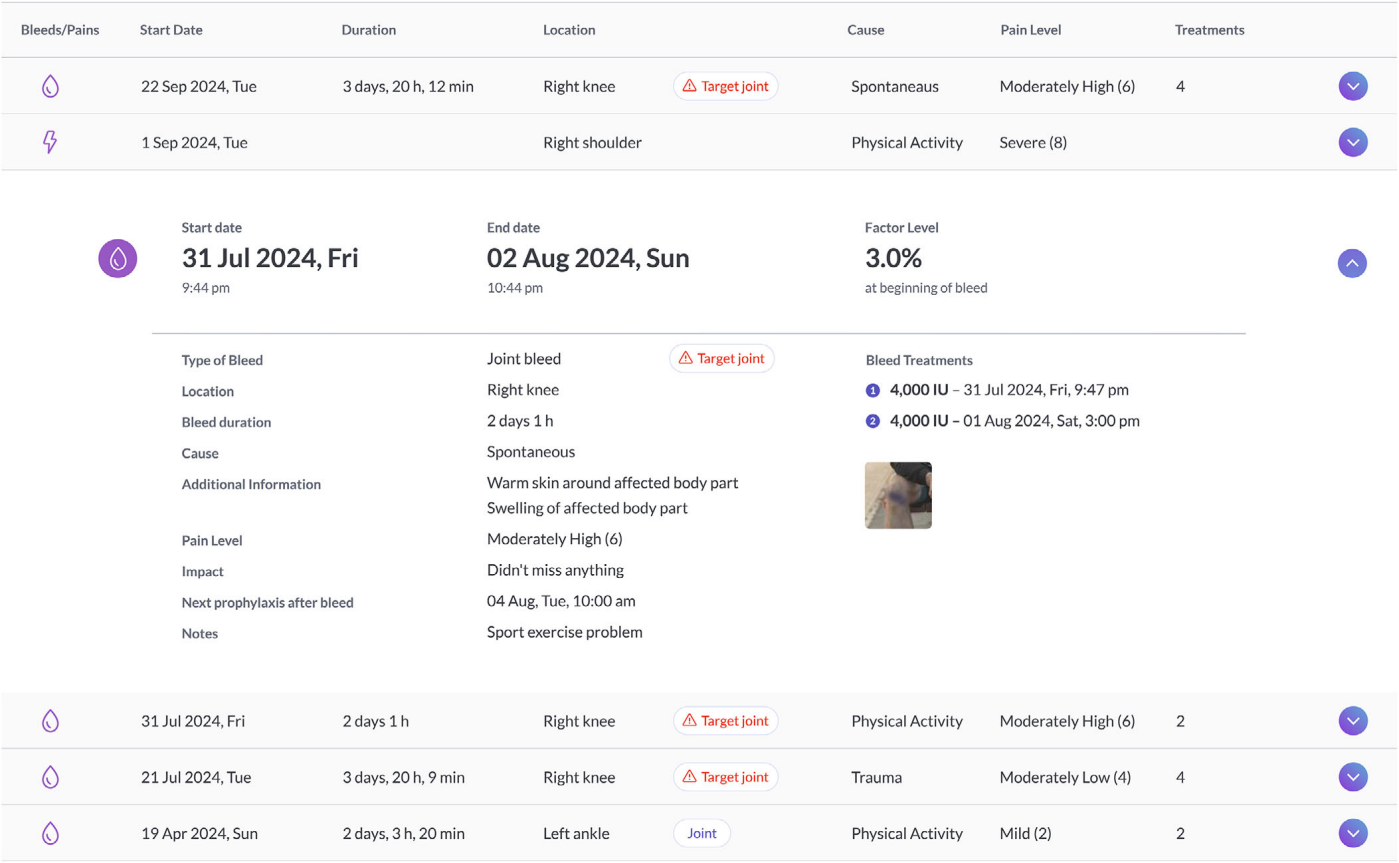

### 2.4 Interconnectivity

To address the unmet need of easily available patient metrics to the community of PLWH and caregivers to build a uniform data pool for comparability of data and common analyses in compliance with data protection and confidentiality, florio HAEMO is designed in such a way that synchronisation with national or international registries and hospital management systems is possible and supported. Connectivity is ensured using an API (application programming interface) based on the health care FHIR (Fast Healthcare Interoperability Resources) standard. This standard allows connection and data exchange of health data in a standardised manner across systems and platforms. Additional bespoke connections to single registries are also possible.

### 2.5 Data Protection

All florio products have been designed in accordance with the *European General Data Protection Regulation* (GDPR), and comprehensive measures have been implemented to protect data based on state‐of‐the‐art technology [32, 33]. Data are stored on servers in the EU, in addition multiple encryption methods are used to ensure data security during data storage and data transfer. This includes encryption of stored data and secure data transmission using end‐to‐end encryption technology. The systems are also regularly tested by external security experts, who probe the systems for vulnerabilities to malicious attacks or accidental data loss. Additionally, the use of non‐identifiable data for research is governed by an external data governance board.

The development of florio HAEMO was sponsored by industry (Swedish Orphan Biovitrum AB [Sobi]). Florio GmbH is an independently operated subsidiary of Sobi. Sobi does not have access to the data collected in florio HAEMO.

### 2.6 Medical Research Based on Aggregated and Anonymised Data

Aggregated or de‐identified data may be used to advance medical research and improve care for PLWH. The access to data analyses of aggregated and de‐identified data is granted for medical research purposes only and governed by an independent external *Data Governance Board* consisting of internationally renowned medical experts and patient association representatives, which are authors of this manuscript. All healthcare stakeholders including the parent company Sobi are subject to the *Data Governance Board* procedures for obtaining access.

## 3 Results and Discussion

### 3.1 florio HAEMO Cohort

**FIGURE 3:**
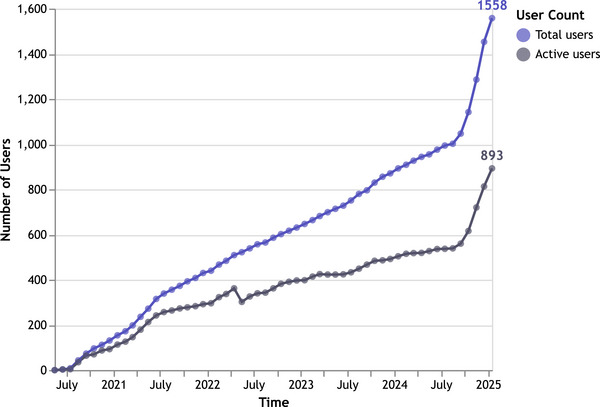
Number of florio HAEMO patient app users over time. Active users are defined as having actively documented in the last 30 days.

Since the launch in March 2020, florio HAEMO has been made currently available in 25 countries and 24 languages. Figure 3 shows the number of users and treatment centres participating over time (cut‐off date: January 15th, 2025). Currently, 1558 PLWH are registered in 121 treatment centres across 20 countries. The majority of users are living with haemophilia A (86%). To date, users have recorded 340,497 injections (including prophylaxis and bleed treatments), 10,858 bleeding events and 2469 pain episodes. All users included so far are on a prophylactic treatment regimen. The cumulative data captured represent 4673.1 patient years.

An overview of first data collected is presented in Table 1.

**TABLE 1:** Overview of first data collected with florio HAEMO (data cut 15.01.2025).

Parameter investigated	Total until January 15th, 2025
Number of active countries	20
Number of active centres (with users)	121
Number of users with logged injections (HA/HB)	1390 (1195/195)
Mean age (min, max; years)	32.2 (1, 87)
Number of injections documented	340,497
Number of bleeding episodes documented (spontaneous)	10,858 (1434)
Number of pain episodes documented	2469
Patient years reported	4673.1

Abbreviations: HA, haemophilia A; HB, haemophilia B.

### 3.2 Initial florio HAEMO Experience

To date, initial experiences with florio HAEMO have been published from two observational studies from Spain and Greece [34, 35, 36] as well as a cross‐sectional survey from six European countries (Croatia, Czech Republic, Hungary, Poland, Slovakia and Slovenia; [30]). Additionally, a survey about the use of different apps in patients with coagulopathies in Spain based on Google forms was conducted in 2021 [21].

Florio HAEMO was reported to be user friendly from almost all participants (97% reported the app to be easy or rather easy to use) in a cross‐sectional survey [30]. In addition, users reported that the app had a positive impact on their living with 78.8% stating that the app was very important or rather important to them [30]. Another survey showed that apps were the preferred registration method, were simple in use, provide reliable and useful records and could promote greater adherence. florio HAEMO was the preferred app by patients due to its estimation of real time factor levels and a more attractive and intuitive design [21].

Another conference abstract reported improvement in outcomes with the use of florio HAEMO in a small Spanish cohort [34]. There was no significant change in adherence to treatment before and after the use of florio HAEMO (median 94% vs. 93%), but a significant decrease in ABR was observed after the use of the application (median 1 [0–8] vs. 0 [0–5.3], *p* = 0.009) [34]. The authors report an increase of PLWH experiencing zero bleeds from 26.1% (*N* = 6) to 56.5% (*N* = 13; *p* = 0.036).

In addition, florio HAEMO is used in several observational studies to capture more granular patient reported information (A‐MORE: NCT04293523, B‐MORE: NCT03901755; JOIN‐us: NCT05856266). A study version based on florio HAEMO is used as data capture in a phase 3b trial FREEDOM (NCT05817812).

These first data illustrate that florio HAEMO is an easy‐to‐use and intuitive app to assist self‐management of haemophilia treatment, adjustment of activities to daily life and analysis of treatment outcomes to further personalise care. [30]. Moreover, preliminary study results in an uncontrolled cohort showed that by the use of florio HAEMO a significant reduction in ABR and an increase in the proportion of PLWH with zero bleeds could be achieved [34]. Further confirmation of this early result is necessary to draw any final conclusions.

## 4 Outlook and Further Considerations

Preventing bleeds, preserving and/or maintaining joint health as well as enabling patients to live a near to normal life is the established new treatment goal in haemophilia. In the era of long‐acting replacement therapies, non‐factor replacement therapies, the availability of gene therapy and ultra‐long factor therapies, monitoring of treatment success and assessing treatment choice based on personal data to define individual goals in life remains key.

PLWH today should be active and strive to achieve the same personal goals as their peers. Understanding individual factor levels in correlation with other parameters as treatment schedule design, physical activities, pain and well‐being may help to further improve planning and allow participation in desired activities while maintaining joint health and avoiding bleeds.

Various studies showed that telehealth‐delivered innovative interventions such as web‐based and mobile apps could improve patients’ adherence to medication and promote independence in disease management within the scope of home therapy for haemophilia [17, 18, 37]. In this context, florio HAEMO allows the collection of contemporaneous data on factor level evolution, treatment adherence, pain levels, well‐being, activity, injection frequency, consumption, information on different treatment regimens and products supporting patients and HCP to take personalised treatment decisions. Importantly, those data can be evaluated in correlation to each other, enabling a new level of data to be reviewed and interpreted.

Initial studies suggest that florio HAEMO is a useful tool, which allows PLWH and clinicians to better manage their condition [30, 32, 33, 34, 35, 36]. In addition, florio HAEMO offers the ability for PLWH on prophylaxis with factor concentrates to share personal disease‐related information with their physicians and optimise their treatment plan and care, allowing potential correlations and adjustments. Data collected over time will help to show the impact of individualised prophylaxis and may support the definition of factor levels required for good bleed and joint protection in a real world setting from daily life to physical activities.

Furthermore, while capturing of contemporaneous data supports treatment decisions on an individual level and individual physical activity, the data collected in florio HAEMO have the potential to also support treatment and care evaluations as well as treatment recommendations on an international level. florio HAEMO offers a useful data set and the interoperability with multiple registries and databases for future considerations to fill the gap of the existing unmet need for a global haemophilia data capture system.

Florio HAEMO is a comprehensive, easy to use platform for PLWH, their caregivers and their health care professionals that offers several unique features such as intuitive tracking of haemophilia related events, correlation of data with real‐time factor levels, activity tracking. Future enhancements to expand collected datasets and improve analysis capabilities for HCPs are planned based on the demands of the haemophilia community. Taken together, collection and correlation of parameters, such as pain and bleeds, as well as factor levels and activity levels, with florio HAEMO has the potential to help to further enhance the personalised care and enable PLWH to live their normal life.

## Funding

The publication concept was developed and discussed by the authors who are members or the data governance board. Participation of the authors in this board was financially supported by the Florio GmbH, Munich/Germany. The authors received no honorarium for the preparation of the publication. Florio GmbH had no influence on the content of the publication. Ms. Andrea Rathmann‐Schmitz, PhD, MEDAHCON GmbH, Bonn/Germany received a reimbursement of expenses from the Florio GmbH for medical writing and editorial activities.

## Ethics Statement

The manuscript describes a medical device without including personal patient data.

## Conflicts of Interest

All authors are members of the data governance board of florio HAEMO. KST is employed by Florio GmbH.

## Data Availability

Not applicable.
